# SARS-CoV-2-Encoded MiRNAs Inhibit Host Type I Interferon Pathway and Mediate Allelic Differential Expression of Susceptible Gene

**DOI:** 10.3389/fimmu.2021.767726

**Published:** 2021-12-23

**Authors:** Youwei Zhu, Zhaoyang Zhang, Jia Song, Weizhou Qian, Xiangqian Gu, Chaoyong Yang, Nan Shen, Feng Xue, Yuanjia Tang

**Affiliations:** ^1^ Shanghai Institute of Rheumatology, Renji Hospital, Shanghai Jiao Tong University School of Medicine, Shanghai, China; ^2^ Institute of Molecular Medicine, Renji Hospital, Shanghai Jiao Tong University School of Medicine, Shanghai, China; ^3^ Department of Hepatobiliary Surgery, Wuxi People’s Hospital Affiliated Nanjing Medical University, Wuxi, China; ^4^ State Key Laboratory for Physical Chemistry of Solid Surfaces, Key Laboratory for Chemical Biology of Fujian Province, Key Laboratory of Analytical Chemistry, and Department of Chemical Biology, College of Chemistry and Chemical Engineering, Xiamen University, Xiamen, China; ^5^ State Key Laboratory of Oncogenes and Related Genes, Shanghai Cancer Institute, Renji Hospital, Shanghai, China; ^6^ Collaborative Innovation Center for Translational Medicine, Shanghai Jiao Tong University School of Medicine, Shanghai, China; ^7^ Center for Autoimmune Genomics and Etiology (CAGE), Cincinnati Children’s Hospital Medical Center, Cincinnati, OH, United States; ^8^ Department of Liver Surgery, Renji Hospital, School of Medicine, Shanghai Jiao Tong University, Shanghai, China

**Keywords:** SARS-CoV-2, COVID-19, microRNA (miRNA), innate immune response, type I interferon pathway, single-nucleotide polymorphisms (SNPs)

## Abstract

Infection of severe acute respiratory syndrome coronavirus 2 (SARS-CoV-2), causing the rapid spread of coronavirus disease 2019 (COVID-19), has generated a public health crisis worldwide. The molecular mechanisms of SARS-CoV-2 infection and virus–host interactions are still unclear. In this study, we identified four unique microRNA-like small RNAs encoded by SARS-CoV-2. SCV2-miR-ORF1ab-1-3p and SCV2-miR-ORF1ab-2-5p play an important role in evasion of type I interferon response through targeting several genes in type I interferon signaling pathway. Particularly worth mentioning is that highly expressed SCV2-miR-ORF1ab-2-5p inhibits some key genes in the host innate immune response, such as IRF7, IRF9, STAT2, OAS1, and OAS2. SCV2-miR-ORF1ab-2-5p has also been found to mediate allelic differential expression of COVID-19-susceptible gene OAS1. In conclusion, these results suggest that SARS-CoV-2 uses its miRNAs to evade the type I interferon response and links the functional viral sequence to the susceptible genetic background of the host.

**Graphical Abstract:**
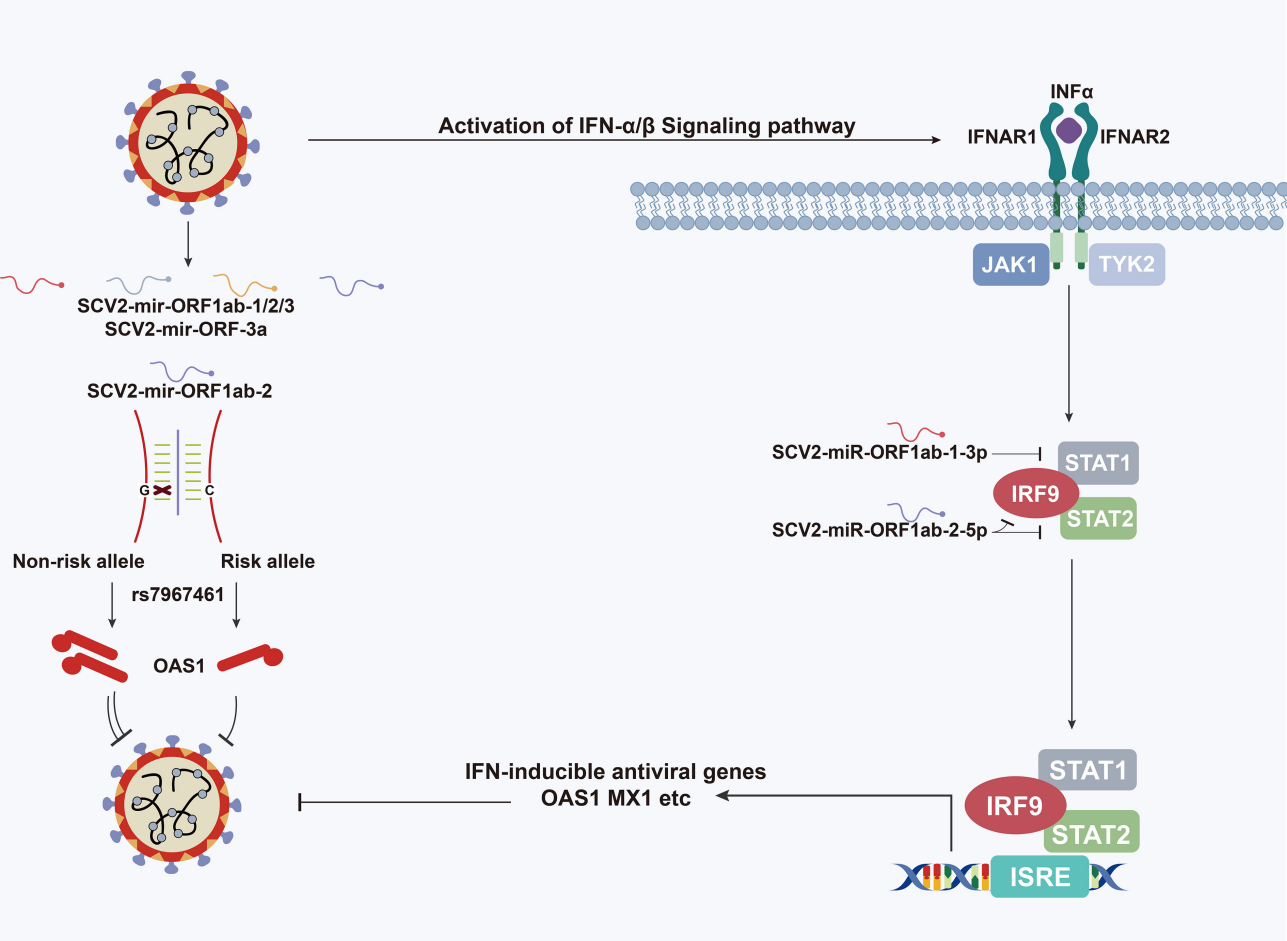
Schematic depiction of the role of SARS-CoV-2-encoded miRNAs in type I interferon pathway.

## Highlights

(1) SARS-CoV-2 encoded four unique miRNAs.

(2) SARS-CoV-2-encoded miRNAs function by regulating host innate immune response to gain a viral advantage.

(3) SARS-CoV-2-encoded miRNA mediated the function of the susceptible site of COVID-19.

## Introduction

The unprecedented worldwide outbreak of coronavirus disease 2019 (COVID-19), caused by severe acute respiratory syndrome coronavirus 2 (SARS-CoV-2), has created a global health emergency. Compared with other two major known coronaviruses, SARS-CoV and Middle East respiratory syndrome coronavirus (MERS-CoV), SARS-CoV-2 has been recognized to be the most contagious, based on the reproductive number R0 (defined as the average number of secondary cases generated per typical infectious case) (1). The initial estimations of the R0 value of SARS-CoV and MERS-CoV were calculated, for China and the Middle East, with R0 median = 0.58 and R0 mean = 0.69, respectively. For SARS-CoV-2, the R0 value associated with the Italian outbreak was calculated with a median point estimate of R0 = 3.1 (2). Recent epidemiological evidence suggests that immune patterns are closely associated with the disease progression of patients infected with viruses (3–5).

The host cells have many receptors that recognize virus elements and induce an innate immune response, such as the activation of the innate immune response, providing the first line of defense against viral infections (6–8). The functional sequences in the viral genome are critical to understanding the spread and evolution of the virus. Discovering and elaborating the functional molecules produced by SARS-CoV-2 are critical to comprehending how the virus replicates and how it escapes the human defense system. Extensive evidence and considerable efforts have revealed that COVID-19 is a systemic disease having the host innate immune response starting when the viral products are recognized by the host cell pattern recognition receptors, including Toll-like receptors and RIG-I-like receptors (9–11). This results in the production of interferon (IFN)-I and other IFN-stimulated genes (ISGs), especially the OAS gene family (OAS1, OAS2, and OAS3), which have been found to degrade a series of the viral genome. Although the type I IFN responses were reported to be significantly activated in most cases, the responses were observed to be impaired in some severely infected patients, especially in association with SARS-CoV-2 infection (12, 13). Coronaviruses have developed mechanisms to antagonize the IFN response to protect themselves against the antiviral effects of the host cells (14). Several researches have been conducted to recognize the critical role of SARS-CoV-2 molecular elements, such as structural and non-structural proteins, in counteracting the IFN signaling pathway immediately after infection and throughout the course of the infection. Recent studies proved that sets of viral proteins are antagonizing the IFN-I signaling: nsp6, nsp13, and ORF6 suppress IRF3 translocation and IRF3/TBK1 phosphorylation, along with the ORF7a mutation to limit viral suppression of the IFN response (15–18). Apart from the abovementioned peptides and proteins, also miRNA-like molecules were reported in many studies as participating in the pathogenic process of virus infection (19). Since the first virus-encoded miRNA was discovered for the human Epstein–Barr virus (EBV), more than 500 viral miRNAs have been reported (20, 21). More specifically, three virus-encoded miRNAs were found by deep sequencing data from the lungs of infected SARS-COV-1 mice, and the inhibition of these miRNAs was observed to significantly reduce the *in vivo* lung pathology, indicating that miRNA produced by a virus is an important potential therapeutic target (22). To date, some bioinformatics analyses have been performed to predict miRNAs encoded by SARS-CoV-2 (23–26). However, the results obtained by different bioinformatics tools are inconsistent, and this is affecting the understanding of the link between SARS-CoV-2 and functional miRNA production.

In this study, we identify four unique miRNAs encoded by SARS-CoV-2 through bioinformatics analysis and small RNA-seq data mining, and we demonstrate how SARS-CoV-2 uses its miRNAs to suppress host innate immune response by targeting several genes in type I interferon signaling pathway. Furthermore, we suggest that SARS-CoV-2-encoded miRNAs may mediate allelic differential expression of COVID-19-susceptible genes. Our findings will stimulate interest in focusing on the correlations between SARS-CoV-2-encoded microRNAs and the host innate immune system and will support the understanding of the molecular basis of genetic susceptibility.

## Method and Materials

### MiRNA Sequencing Data Collection and Preparation

Complete genome data (SARS-CoV-2 isolate Wuhan-Hu-1) and relative annotation of the virus were obtained from the National Center for Biotechnology Information (NCBI) (NCBI Reference Sequence MN908947.3). High-throughput small RNA sequencing data were downloaded from the Gene Expression Omnibus (GEO) database (https://www.ncbi.nlm.nih.gov/geo/query/acc.cgiacc=GSE148729). Sequencing reads were trimmed by cutadapt v2.9 (27) in two passes. First, the parameter (cutadapt -a AGATCGGAAGAGC -u 3 -q 10 -o trimed.fastq non-trimed.fastq) was used to cut the TruSeq adaptor. In the second pass, poly(A)-tails (cutadapt -a A(2) -minimum-length 18 -maximum-length 24 -o clean.fastq trimed.fastq) were trimmed. After quality control, the length distribution of clean miRNA was analyzed.

### Discovery of Candidate Viral-Derived MiRNAs

Novel miRNA discovery was performed using modules of miRCat (28) and MiRmat (29) prediction software combined with the exploration of miRNA-seq coverage profile. First, a miRNA sequencing reads alignment analysis was conducted. Bowtie v1.2.2 (30) was used to map the trimmed small RNA sequencing data to the SARS-CoV-2 genome with the non-standard parameters (-q -n 0 -e 80 -l 18 -a -m 5 –best –strata). Second, with respect to the prediction software, a list of all candidate mature miRNAs and their genomic coordinates of 5p or 3p sites was obtained from the results of the miRCat (28) algorithm. The parameter of miRCat was set to default (-srna_file sequencing file -genome path to SARS-CoV-2 genome -output_directory path to output directory) with the file of default_mircat_params.cfg UEA sRNA Workbench v 4.7 chosen as reference. Then, the miRmat (29) algorithm was applied to find Dicer and Drosha restriction sites on the viral genome, thereby confirming the region of the precursor and mature miRNA.

### Viral MiRNA Conservation Analysis

The SARS-CoV-2-encoded miRNAs (SCV2-miRNAs) were first compared with human or other viral miRNAs deposited in the miRbase platform by using the Basic Local Alignment Search Tool (BLAST) program (http://www.mirbase.org/search.shtml). Then, SimPlot v3.5.1 (31) was employed to conduct a conservation analysis between SCV2-miRNAs and miRNAs derived from other coronaviruses. More specifically, a window sliding analysis was performed to determine the changing patterns of sequence similarity between the query sets (other coronaviruses) and the reference sequence (SARS-CoV-2).

Phylogenetic analysis was then performed by extracting sequences of the predicted 4 pre-miRNAs. Homologies in 28 related coronavirus genomes (32) of these 4 sequences (queries) were obtained using BLASTn. For each query, the hits with e-value greater than 0.05 or alignment length shorter than 60% with respect to the reference sequence were discarded. A multiple sequence alignment of these homologies, consisting of the 4 pre-miRNAs sequences and the filtered BLASTn hits, was generated using MAFFT v7.471 (33). Phylogenetic trees were built using online tools in the ATGC platform (34) with the GTR substitution model and 1,000 bootstraps. ETE 3 (35) was applied to visualize the phylogenetic tree.

### Association Analysis of Variants in SCV2-MiRNAs With COVID-19 Severity

All 544 sequences (207 mild and 337 severe) with a defined patient status, and satisfying the prerequisites of having complete genomes (>29,000 nt) and high coverage (sequences with >5% Ns were filtered), were manually collected from GISAID (36) (from October 10, 2020, to February 26, 2021). The patients were classified according to their status, as follows: “mild” patient when the metadata directly described the status as “mild” or “moderate,” and “severe” when the metadata included the words like “ICU”, “severe”, and “deceased”. Multiple sequence alignment was conducted by MAFFT v7.471 (33). All the loci of the SCV2-miRNA region were extracted, and the genome-wide association analysis was performed with an R package called TreeWAS (37). The required maximum likelihood phylogenetic tree was prepared by IQ-TREE v2.0.3 (38) with the GTR+I+R model. The number of sites simulated to estimate the null distribution was 500 times the extracted locus, and the p-value for association tests was set to 0.05.

### RNA Sequencing Data Analysis

After the read counts table of total RNA-seq was downloaded from the GEO platform (GEO: GSE148729, GSE148729_Calu3_totalRNA_readcounts.tsv.gz), the expression analysis was performed using the edgeR algorithm v3.12 (39) by comparing 24-h treat infections and mock groups. For each gene transcript, the summarized FPKM values, in both the 24-h infected and mock groups, were required to be greater than or equal to 45 (FPKM Treat 24-h A/B + FPKM Mock 24-h A/B). The differentially expressed genes (DEGs) were defined as genes with an absolute fold-change of greater than 1.5 and a p-value of less than 0.05. The DEGs were then subjected to gene set enrichment analysis through the EnrichR (40) platform (https://maayanlab.cloud/Enrichr/). In terms of function annotation, the results of pathway analysis were obtained from the Bioplanet (41) pathway analysis module.

### Prediction of MiRNA Targets Involved in Enriched Pathway

Two algorithms were used for miRNA target prediction: RNAhybrid (42) and miRanda (43) with the non-standard parameters (RNAhybrid: RNAhybrid -c -f 2,7 -e -15 -s 3utr_human -t reference.fasta -q miRNA-seq.fasta > result.txt, miRanda; miranda reference.fasta -sc 140 -en -15 -out result.txt). Target prediction was performed on all RefSeq hg38 mRNA transcript isoform sequences (including the 5′-UTR, CDS, and 3′-UTR).

### Cell Culture, Transfection, and Stimulation

HEK293T cells were obtained from the Cell Bank, Shanghai Institutes for Biological Sciences, Chinese Academy of Sciences, and grown in Dulbecco’s modified Eagle’s medium (Gibco) containing 10% fetal bovine serum (Gibco). All cells were maintained at 37°C with a 5% CO_2_ atmosphere. Plasmids or RNAs were transfected at the final concentration of 200 nM into cells with Lipofectamine RNAiMAX (Invitrogen) or Neon Transfection system according to the manufacturer’s instructions. Plasmids and transfection reagents were diluted with Opti-MEM medium (Gibco) and incubated at room temperature for 10 min, after being gently mixed. Then, the transfection mixture was added to the cell culture. Under 1,245 pulse voltage condition, 10 pulse width, and 3 pulse number, electroporation in 100-µl volumes was carried out, as described in the Neon instruction manual (Neon Transfection System MPK5000). Cells were harvested 48 h after transfection. Type I IFN (PBL) was added at the final concentration of 200 units/ml.

### Quantitative Reverse Transcription-PCR

Total RNA was extracted using TRIzol (Ambion) in accordance with the manufacturer’s instructions, and cDNA was synthesized by PrimeScript RT Reagent kit (Takara) or miRNA First Strand Synthesis Kits (Takara). Then, DNA amplification and quantification were performed through real-time PCR with SYBR Premix Ex Taq™ kit (Takara) in QuantStudio™ 7 Flex Real-Time PCR System (Applied Biosystems). The target genes and miRNA relative expression levels were calculated using the 2^−ΔΔCt^ method normalized to GAPDH or U6. The sequences and Primer of SCV2-miRNAs and the mock/control microRNAs are provided in **Supplementary File 1**.

### ISRE Reporter Gene Assay

The ISRE (IFN-stimulated response element) reporter plasmid and Renilla plasmid were co-transfected with SCV2-miRNA into HEK293T cells and seeded in a 96-well plate. One day after transfection, type I IFN (PBL) at the final concentration of 200 units/ml was added to stimulate cells for another 24 h. Then, the cells were harvested, and the cell lysis solution was added into a 96-well black flat-bottom microplate (Greiner Bio-one). Their luciferase activities were measured on a CENTRO XS3 LB 960 luminometer (Berthold) using Dual-Luciferase Reporter Assay System (Promega). The Firefly : Renilla luciferase ratio for each well was calculated later. All experiments were performed in triplicate, each being repeated at least three times.

### Western Blotting

HEK293T cells were seeded at 4 × 10^5^/well in a 6-well plate and transfected with SCV2-miRNA at the final concentration of 200 nM for 48-h incubation. Then cells were lysed with radioimmunoprecipitation assay (RIPA) buffer (Pierce, Thermo Scientific) and supplemented with protease inhibitor cocktail (Pierce, Thermo Scientific). The cell protein was loaded into sodium dodecyl sulfate–polyacrylamide gel electrophoresis (SDS-PAGE) and blotted with the appropriate antibodies. Band signals were visualized with a SuperSignal West Pico kit (Pierce, Thermo Scientific). The antibodies used were GAPDH rabbit mAb (horseradish peroxidase (HRP) conjugate) and STAT1 rabbit mAb. All the antibodies were purchased from Cell Signaling Technology. The primary antibodies were diluted at a ratio of 1:3,000 (GAPDH) and 1:200 (STAT1 and STAT2). The secondary antibody was diluted at a ratio of 1:5,000.

### Acquisition of Single-Nucleotide Polymorphism Sites in the Targets of SCV2-MiRNAs

First, single-nucleotide polymorphisms (SNPs) were searched on the SCV2-miRNAs target genes deposited in dbSNP150 (44), and the ANNOVAR (45) platform was used to collect the information on these polymorphic sites. The binding pairs of miRNA–SNP were extracted by using the sequence of 35-bp length previously obtained and centered on the SNP site, with a threshold of minimum free energy (MFE) of −15.0 kcal/mol. Then, the interaction between different allele genes and SCV2-miRNAs was analyzed through the software of RNAhybrid (42), and the energy value change ΔG > 1 kcal/mol between the different alleles of SNP interaction with SCV2-miRNA was set as the criterion.

The result tables of COVID-19 Host Genetics Initiative (46) meta-analyses round 4 (https://www. covid19hg.org/results/) were obtained. The raw data of three phenotypes were chosen for further analysis: COVID-19 vs. population; severe respiratory confirmed COVID-19 vs. population; and hereafter hospitalized COVID-19 vs. population. The commonly accepted threshold of p-value < 1E−08 as a significant criterion was used to select SNPs for further analysis. Additionally, linkage disequilibrium (LD) linkage sites of each COVID-19-associated SNP were figured out with the set of parameter r^2^ at 0.8 (high linkage parameter), and EUR subpopulation was chosen as reference panel through the HaploReg v4.1 (47) platform.

### Single-Nucleotide Polymorphism Reporter Gene Assay

The sequences including different alleles of rs7967461 were cloned (Ref : TTAGTGAACATGCGGTGAATTTGCAACAGACAAGAGGAGCCTCATTATCCTATAGTTTCCAGGTTGCTTAG; Alt : TTAGTGAACATGCGGTGAATTTGCAACAGACAAGACGAGCCTCATTATCCTATAGTTTCCAGGTTGCTTAG) into the psiCHECK2 reporter plasmid. All constructs were confirmed by sequencing. Then, SCV2-miR-ORF1ab-2 and psiCHECK2 reporter plasmids were transfected into HEK293T cells by lipo3000 (Invitrogen) in each well of a 96-well plate for 24-h incubation. The next experimental step was the same as ISRE Reporter Gene Assay.

### Quantification and Statistical Analysis

GraphPad Prism 7 (48) was chosen for data analysis. The results are presented as the standard error of the mean (SEM). Comparisons of groups were conducted using two-tailed Student’s t-tests or one-way ANOVA, and p-value < 0.05 was considered as statistically significant, unless otherwise specified.

## Results

### Identification of SARS-CoV-2-Encoded MiRNAs

**Figure 1:**
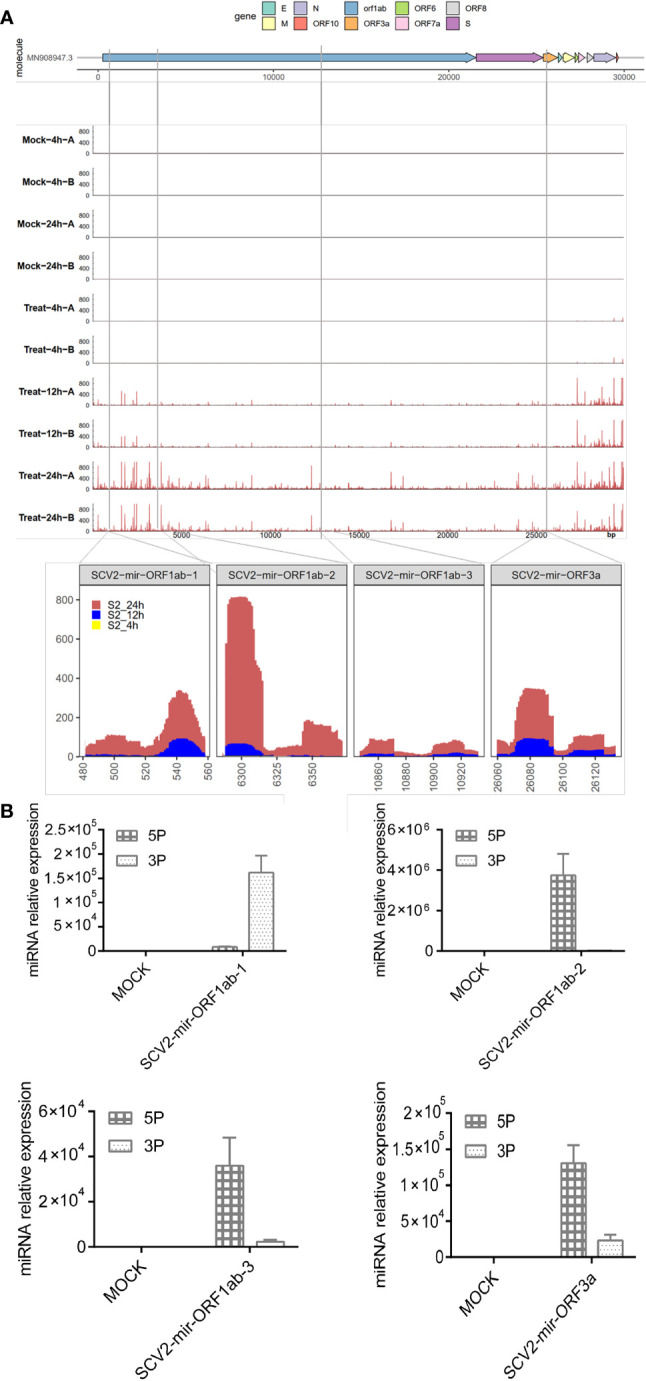
Identification of miRNAs encoded by SARS-CoV-2. **(A)** Top: the names of viral genes and the genome positions (nt) are displayed with details of the genomic regions, including the four predicted pre-miRNAs, indicated by black stripes. Center: small RNA-seq coverage map of the small RNA sequences aligning with the SARS-CoV-2 genome. Bottom: detailed depiction of small RNA-seq coverage across the four pre-miRNAs encoded by SARS-CoV-2. **(B)** The real-time quantitative PCR of SARS-CoV-2-encoded mature miRNA. Predicted SCV2-miRNA precursors were synthesized and electroporated into HEK293T, which were seeded onto 24-well plates for 48 h. Relative quantification of SCV2-miRNA 5P and 3P expressions was estimated through qRT-PCR. Data are expressed as mean ± SEM of three independent experiments.

According to the summary of the GEO database for small RNA sequencing series (GEO accession number: GSE148729), there was a total of 10 libraries (four in the mock stage and six in the infectious stage) representing two stages of infection development: Mock and Treat. To clarify whether SARS-CoV-2 generated small RNAs, we aligned small RNA sequencing reads to the SARS-CoV-2 genome. We found that SARS-CoV-2 produced small RNAs that were accumulating after the virus infection, especially in the 24-h infection treatment group (**Figure 1A**). Next, we used bioinformatics tools (modules of miRCat (28) and miRmat (29)) combined with small RNA sequencing data to predict SARS-CoV-2-encoded miRNAs. Only reads with a length between 18 and 24 nt mapped to the viral genome with a mismatch of 0 are considered for real miRNA. Four miRNA precursors with stem-loop structure were derived from ORF1ab and ORF3a regions of the SARS-CoV-2 genome (**Figure 1A**), and the mature miRNAs were named SCV2-miR-1ab-1-3p, SCV2-miR-1ab-2-5p, SCV2-miR-1ab-3-5p, and SCV2-miR-3a-5p.

To test whether pre-SCV2-miRNAs (precursors of SCV2-miRNA) are really processed into mature SCV2-miRNAs, pre-SCV2-miRNAs were synthesized and transfected into HEK293T cells. After 48 h, these cells were harvested, and the profile of mature SCV2-miRNAs was quantified using qRT-PCR (49). As shown in **Figure 1B**, the pre-SCV2-miRNAs could be literally processed into mature SCV2-miRNAs. Consistent with the sequencing data, SCV2-miR-1ab-2-5p was observed to be highly expressed in cells.

### Homology and Mutation Analysis of SCV2-MiRNAs

**Figure 2:**
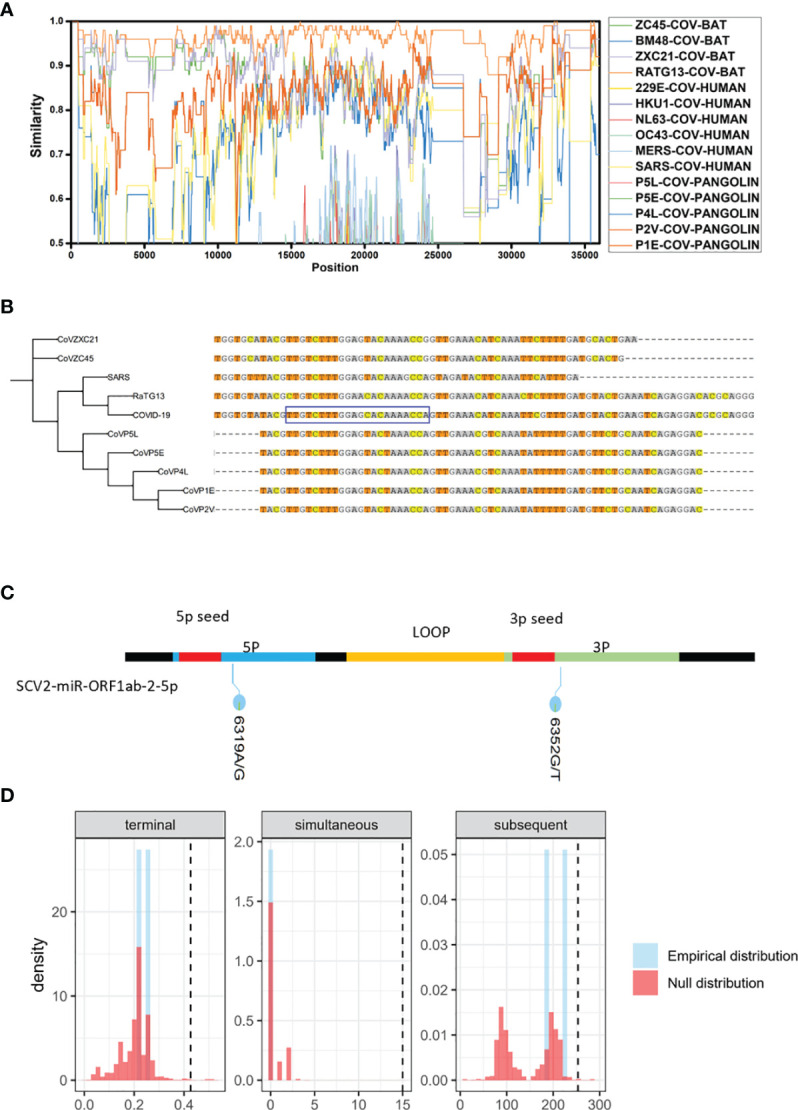
Analysis of SARS-CoV-2 sequence homology and mutation. **(A)** Sliding window analysis of changing patterns of sequence similarity between human SARS-CoV-2 and query sequences sets (human, pangolin, and bat coronaviruses), displayed in different colors. The image above highlights the similarities between SARS-CoV-2 and other human coronaviruses. Names of query sequences are located vertically on the right side of the graphical analysis. **(B)** Phylogenetic trees of SCV2-mir-ORF1ab-2 and related sequences of other coronaviruses. One thousand bootstraps replicates were applied. The sequence in the blue rectangle refers to the SCV2-miR-ORF1ab-2-5p. **(C)** Positions of observed single-nucleotide variations (6319A→G 6352G→T) on the SCV2-miR-ORF1ab-2-5p and their corresponding frequencies. **(D)** Results from three statistical tests integrated into TreeWAS (Terminal test, Simultaneous test, and Subsequent test). TreeWAS was used to identify genetic variations in the SCV2-mir-ORF1ab-2 region potentially associated with COVID-19 severity. The three null distributions of each test score from the simulated data are shown in red, and the thresholds are illustrated with dotted black vertical lines. The three distributions of the two single-nucleotide variations tests scores, identified from the real sequencing data, are shown in blue and gray.

To illustrate the homology of SARS-CoV-2, we first conducted a sliding window analysis of sequence similarities among human, pangolin, and bat coronaviruses. The results are show that bat coronavirus RaTG13 has the highest similarity (0.962) with SARS-CoV-2, followed by bat coronavirus ZXC21 (0.865) and pangolin coronaviruses (P5L, P5E, P4L, P2V, and p1E). Also, human coronavirus SARS-CoV-1 has a remarkable resemblance with SARS-CoV-2 (0.740–0.837) (**Figure 2A**). The information regarding strains applied during the analysis is summarized in **Supplementary File 2**.

We then analyzed the homologous and evolutional relationships of SCV2-miRNAs and their precursors. We found that only human coronavirus SARS-CoV-1 and coronavirus RaTG13 in bat species have homologous sequences with both pre-miRNA and miRNA in SCV2-miR-ORF2ab-2 regions (**Figure 2B**). Considering that the SARS-CoV-1 is known as human coronavirus and that it is highly homologous with SCV2-miR-ORF2ab-2 regions, it may share conserved miRNAs with SARS-CoV-2. However, the same bioinformatics prediction software that predicted the vmiRNA encoded by SARS-CoV-2 could not detect the putative miRNA in this region. In addition, we used the BLAST program (50) to further explore whether the SCV2-miRNAs are conserved with human miRNAs and other viral miRNAs, deposited in miRBase. We found that there is no significant similarity between SCV2-miRNAs and other known human miRNAs or other viral miRNAs. These results demonstrate that SARS-CoV-2 encodes its own specific miRNAs.

To detect the mutation on the virus-encoded miRNA, we manually collected from GISAID the SARS-CoV-2 sequencing data with unambiguous patients’ status (**Supplementary File 3**) and called SNPs in miRNA regions. However, we found only two genomic variants in SCV2-mir-ORF1ab-2 with a mutation frequency of 6/538 (**Figure 2C**). Furthermore, we used the R package TreeWAS (37) and performed a genome-wide association study (GWAS) analysis to identify whether these two variations were associated with the COVID-19 severity. Since the score distribution of 6319G/A and 6352T/G shown in blue does not pass the black vertical lines (which determines whether the mutation can significantly contribute to the pathogenicity of the virus in “Terminal” (left), “Simultaneous”, and “Subsequent” test (**Figure 2D**), we can conclude that the genetic variations, which are not located in the seed-sequence region of SCV2-miR-ORF1ab-2, are not significantly associated with the disease severity.

### Functional Analysis of SCV2-MiRNAs in Type I Interferon-Related Pathway

**Figure 3:**
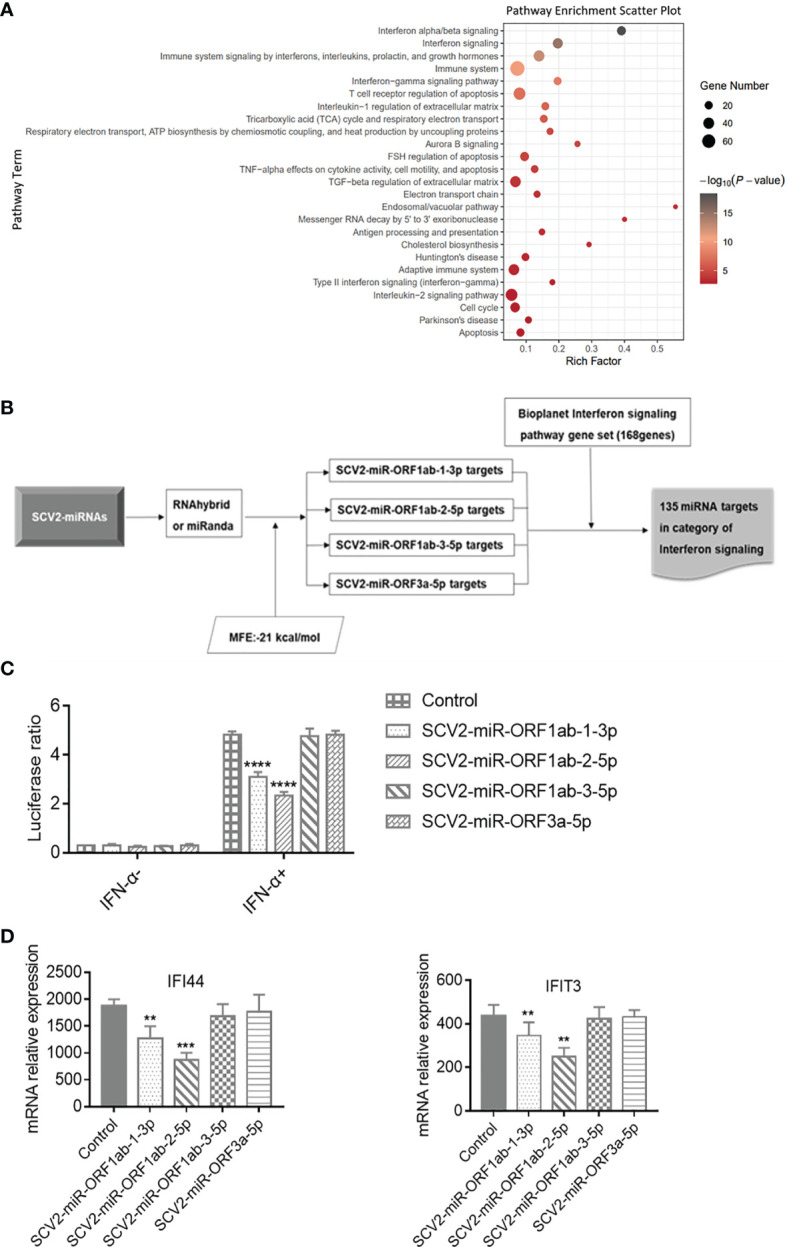
Functional analysis of SCV2-miRNAs. **(A)** The scatter plot showing the pathway enrichment of differentially expressed genes (DEGs) in RNA-seq experiments of Calu-3 cells infected with the virus infection versus mock at 24 h. Bubble color is proportional to gene number in −log10 (p-value) and size. **(B)** Flowchart of the screening process for the genes targeted by SCV2-miRNAs in type I interferon pathway. **(C)** Effect of SCV2-miRNAs on the activation downstream of type I interferon (IFN) assessed by IFN-stimulated response element (ISRE)–Luc reporter system. HEK293T cells were co-electroporated with SCV2-miRNAs, ISRE Firefly luciferase reporter plasmid, and Renilla luciferase control plasmid. After 24 h, the cells were stimulated with type I IFN (200 U/ml) for 24 h, and luciferase activity in cell lysates was detected at the indicated time points. **(D)** Relative expression level of interferon-stimulated genes (ISGs) was measured in HEK293T cells for 200 U/ml of type I IFN in 24-h stimulation and analyzed by qRT-PCR. Data are expressed as mean ± SEM of three independent experiments. p-Values were analyzed with two-tailed unpaired t-test. **p < 0.01, ***p < 0.001, ****p < 0.0001.

There have been many investigations into the host gene expression after SARS-CoV-2 infection (51–53). To this extent, differential expression analyses were performed on the basis of RNA-seq data, comparing Treat and Mock groups at 24 h upon infections and identifying 1,279 DEGs. Function annotation revealed that these DEGs are enriched mainly in the immune system-related pathway (**Figure 3A**), and three of the most enriched categories were all found to be associated with IFN response to virus resistance: alpha/beta signaling, IFN signaling, and Immune system signaling by IFNs, interleukins, prolactin, and growth hormones. Furthermore, the term enrichment of the immune system accounted for 125 enriched DEGs in total, covering the genes of the three abovementioned enriched signaling pathways (see **Supplementary File 4**).

Up to now, there have been studies evidencing that SARS-CoV-2 exhibits multiple strategies to counteract the innate immune response (12–14). To determine whether SCV2-miRNAs were involved in evasion of type I IFN response, we first predicted the targets of SCV2-miRNAs by using RNAhybird (42) and miRanda (43) software. Then, we filtered the above target genes for the IFN signaling pathway gene set obtained from the Bioplanet (41) database. A total of 135 IFN pathway-related genes are the potential targets of SCV2-miRNAs, and this indicates that SCV2-miRNAs may regulate type I IFN signaling (**Figure 3B** and **Supplementary File 5**).

To assess the role of SCV2-miRNAs in the IFN-α/β triggered signaling pathway, we transfected SCV2-miRNAs with ISRE Firefly luciferase reporter plasmid and Renilla luciferase control plasmid into HEK293T cells. We then stimulated the cells with type I IFN (200 U/ml) for 24 h. Subsequently, the Firefly and Renilla luciferase activity was measured by Dual-Luciferase Reporter Assay System (Promega), and the Firefly : Renilla luciferase ratio for each well was calculated later. As shown in **Figure 3C**, SCV2-miR-ORF1ab-1 and SCV2-miR-ORF1ab-2 significantly reduced the activity of ISRE, while SCV2-miR-ORF1ab-3 and SCV2-miR-ORF3a had no apparent effect. Coincident with the results of the ISRE reporter gene assay, SCV2-miR-ORF1ab-1 and SCV2-miR-ORF1ab-2 were also observed to suppress the ISG expression in HEK293T cells transfected with SCV2-miRNAs (**Figure 3D**). These data suggest that SARS-CoV-2 employs its miRNAs to inhibit the host IFN response.

**Figure 4:**
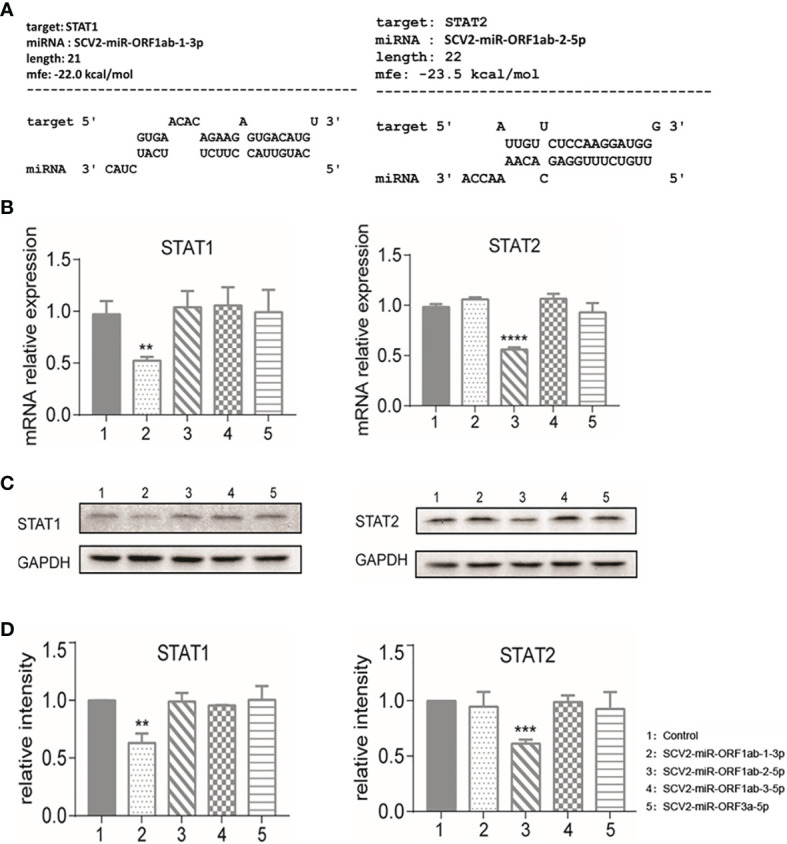
Identification of STAT1 as the target of SCV2-miR-ORF1ab-1-3p and STAT2 as the target of SCV2-miR-ORF1ab-2-5p. **(A)** Schematic presentation of the potential SCV2-miR-ORF1ab-1-3p binding site in the 3′-untranslated region (3′-UTR) of STAT1 and the potential SCV2-miR-ORF1ab-2-5p binding site in the 3′-UTR of STAT2. **(B)** Quantitative PCR analysis of STAT1 and STAT2 expression in HEK293T cells 48 h post-transfection of different SCV2-miRNAs versus control. Histograms show fold changes in mRNA expression with respect to the controls after normalization with the housekeeping gene GAPDH. **(C)** Immunoblot analysis of STAT1 and STAT2 expression in HEK293T cells 48 h post-transfection of different SCV2-miRNAs versus control. **(D)** Protein band intensity quantitatively assessed by ImageJ software. Data are representative of three independent experiments. p-Values were analyzed with two-tailed unpaired t-test. **p < 0.01, ***p < 0.001, ****p < 0.0001.

Using RNAhybrid software for bioinformatics prediction, STAT1 and STAT2 were found to be potentially targeted by SCV-miRNAs (**Figure 4A**). To detect the expression profile of SCV2-miRNAs targets in type I IFN pathway, the SARS-CoV-2-encoded miRNA was overexpressed by transfecting the SCV2-miRNAs into HEK293T. As a result, six genes were observed to be effectively inhibited by two SCV2-miRNAs through qRT-PCR, while seven genes were observed to have little or no inhibition effect (**Figure 4B** and **Supplementary File 6**). Specifically, STAT1 was inhibited by SCV2-miR-ORF1ab-1, and STAT2, OAS1,OAS2, IRF7, and IRF9 were inhibited by SCV2-miR-ORF1ab-2.

Among these targets, STAT1 and STAT2 were commonly recognized as critical factors in producing downstream ISGs in JAK–STAT pathway. In this respect, we further verified the profile of STAT1 and STAT2 related to their protein expression level by Western blotting. From the band signals, it could be concluded that the protein expression level of STAT1 and STAT2 is obviously affected by SCV2-miR-ORF1ab-1 and SCV2-miR-ORF1ab-2 (**Figures 4C, D**). This is consistent with the results of the relative expression level of mRNA (**Figure 4B**).

### SCV2-MiRNA Mediated Allelic Differential Expression of Susceptible Gene

**Figure 5:**
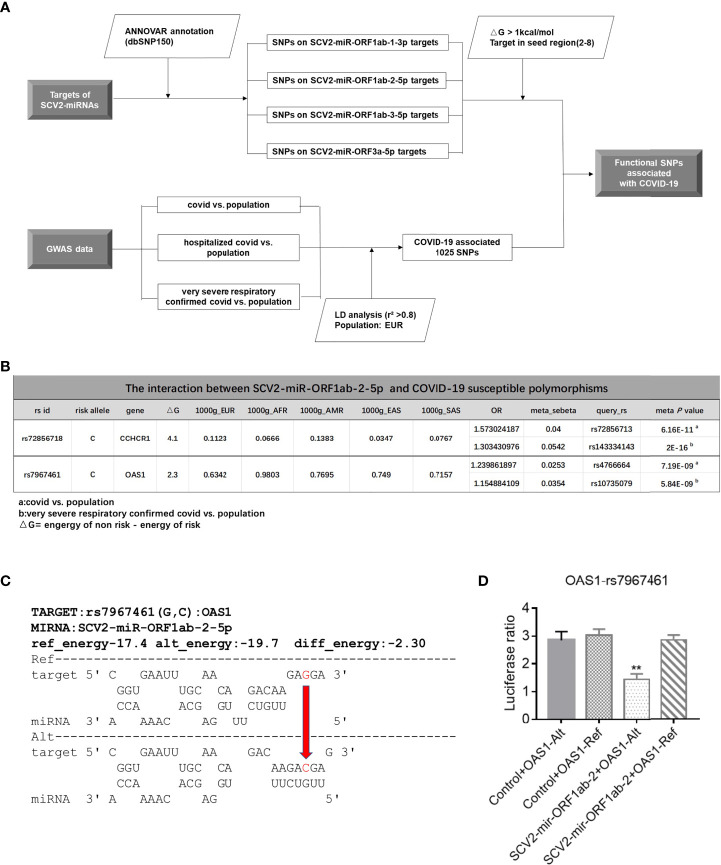
Allelic differential expression of susceptible gene mediated by SCV2-miRNAs. **(A)** Flowchart of the screening process of interaction between SCV2-miRNAs and susceptible polymorphisms sites associated with COVID-19. **(B)** Graphic table representing the information that COVID-19-susceptible polymorphisms significantly altered SCV2-miR-ORF1ab-2-5p interaction with its target. **(C)** The schematic diagram displays the energy change between the two alleles of rs7967461 (G→C) interacting with SCV2-miR-ORF1ab-5p. The value of energy change is depicted above the dotted black horizontal lines. The red arrow indicates the base alteration from Ref-type to the Alt one. **(D)** Variation in the OAS1 3′UTR differentially affects the reporter gene’s expression. The sequences containing OAS1-rs7967461 single-nucleotide polymorphism (SNP) allele (78 bp) were cloned into psiCHEK2 reporter plasmid. HEK293T cells were co-electroporated with SCV2-miR-ORF1ab-2 and SNP reporter plasmid. Forty-eight hours later, luciferase activity in cell lysates was detected. Data are expressed as mean ± SEM of three independent experiments. p-Values were analyzed with two-tailed unpaired t-test. **p < 0.01.

Several studies performed GWAS analysis and found that multiple genetic factors could contribute to the severity of COVID-19 (54–56). However, the underlying biological mechanisms of these SNPs have been mostly unclear. Since the genetic variations in miRNA target sites were reported to influence the miRNA-mediated regulatory functions (57–59), we investigated whether there are SNPs located in SCV2-miRNA targets, and we screened out SNPs with the energy value change ΔG > 1 kcal/mol between the SCV2-miRNA–SNP interaction pairs (**Figure 5A** and **Supplementary File 7**). As shown in the flowchart of **Figure 5A**, we first obtained a group of SNPs in the targets of SCV2-miRNAs. Although most of them are rare mutations with very low frequency, there are also several common mutations. Several previously reported GWAS analyses have identified some common mutations associated with COVID-19 (60), and we have obtained 1,025 susceptible polymorphisms through LD analysis (**Supplementary File 8**). Interestingly, the different alleles of susceptible rs72856718 and rs7967461 may affect the interaction between SCV2-miRNA and target genes (**Figure 5B**). In particular, rs7967461 located in OAS1, which had been reported to degrade a series of the viral genome, would have required a more in-depth study to understand and explain its pathogenic mechanism. Additionally, the interaction predicted by the RNAhybrid (42) algorithm between SCV2-miR-ORF1ab-2-5p and OAS1 including the risk allele of rs7967461 contributes to a more stable interaction compared with the non-risk allele (**Figure 5C**).

To determine whether rs7967461 influences SCV2-miR-ORF1ab-2-5p binding activity, we cloned the fragments containing different alleles of rs7967461 of OAS1 into the 3′UTR region of a reporter gene and then transfected it with or without SCV2-miR-ORF1ab-2-5p into HEK293T cells. After 48 h, we used the dual-fluorescence reporter gene system to analyze the different effects of rs7967461 alleles on luciferase activity under the presence or absence of SCV2-miR-ORF1ab-2-5p. Through the dual-luciferase reporter gene assay, we confirmed that the risk allele of rs7967461 in combination with SCV2-miR-ORF1ab-2-5p led to repressed dual-luciferase gene expression (**Figure 5D**). Our results demonstrate that SCV2-miRNAs could mediate the allelic differential expression of COVID-19-susceptible genes.

## Discussion

Soon after the outbreak of COVID-19, scientists quickly read the sequence of the SARS-CoV-2 genome RNA, which consists of about 30,000 bases. It is critical to understand the life cycle and pathogenicity of SARS-CoV-2 through the functional genetic information that it carries. In this study, we delineate that SARS-CoV-2 uses the host cellular system to generate miRNAs and evade the host immune response.

It is well known that the IFN response is the most important antivirus immune response of the host cells. IFN-I, as well as other ISGs, is produced to form the first line of defense against SARS-CoV-2 (6–8). Understanding the molecular mechanisms of interaction between the virus and its host is the key to comprehending COVID-19 pathogenesis. Although the genome sequence of SARS-CoV-2 exhibits almost 80% identity with SARS-CoV and 50% identity with MERS-CoV, SARS-CoV-2 has been recognized as the most contagious (2). SARS-CoV-2 deploys multiple proteins, highly conserved with SARS-CoV-1, to shut down IFN signaling and dampen innate immune responses (61). This study provides evidence that SARS-CoV-2 can use its own elements for immune evasion. Two unique SCV2-miRNAs were recognized to inhibit the key transcription factors (IRF7, STAT1, STAT2, IRF9, etc.), which play a key role in the production of IFN-I (IFN-α/IFN-β) and the activation of the IFN-I signaling pathway. Our observations suggest that SCV2-miRNAs are new players in suppressing transcriptional activation of antiviral ISGs, which finally supports the virus in establishing a more friendly environment inside the host cells. The interaction between SCV2-miRNAs and host mRNAs also explains the greater infectivity of SARS-CoV-2 compared with other coronaviruses.

Most miRNA-like small RNAs have been reported to be associated with Argonaute proteins (AGO1/2/3/4). The small RNAs are incorporated into AGOs to guide sequence-specific gene silencing by base-pairing with target RNAs, either transcriptionally or post-transcriptionally. MiRNA-like small RNAs can be discovered by examining AGO-interacting small RNAs (62–64). We noticed that many small RNAs, induced by SARS-CoV-2 infection, correspond to the SARS-CoV-2 genome (**Figure 1A**). It is worth mentioning that SARS-CoV-2 may produce some miRNA-like molecules not identifiable, as prediction methods could generate false-negative results. Therefore, the capacity of SARS-CoV-2 to encode functional miRNA-like effectors is needed to systematically explore through analysis of AGO-associated small RNAs by deep sequencing in cells infected with SARS-CoV-2 in further study.

Our study revealed that host genomic variations within SCV2-miRNA target sites may introduce the changes in miRNA–mRNA interaction, which will result in allelic differential expression of target genes and may correlate with individual susceptibility to COVID-19. Our analysis showed that SCV2-miR-ORF1ab-2-5p could target IFN-induced antiviral enzymes OAS1 and OAS2. The more stable interaction between SCV2-miR-ORF1ab-2-5p and the pathogenic risk-allele rs7967461(C) of OAS1 will lead to low OAS1 expression, which can, in turn, be advantageous to the virus. Moreover, rare mutation (rs202081642) in HMGB1 gene, one of the intriguing factors of SARS-CoV-2 infection and sustained inflammation (65, 66), is predicted to be the target of SCV2-miR-ORF3a-5p. The rs202081642 (G→A) polymorphism could weaken the binding ability of SCV2-miR-ORF3a-5p with HMGB1 mRNA, which represents the pathogenic risk of inflammatory storm during SARS-CoV-2 infection. Above all, functional polymorphisms in SCV2-miRNAs targets could represent a valuable starting point to assess the COVID-19 disease risk.

Although our research refreshes the concept of SCV2-miRNAs possibly participating in the pathogenic process of COVID-19, this study has some limitations. First, due to the limitations of experimental conditions, our team cannot obtain effective strains of SARS-CoV-2 to infect cells. It is not confirmed that the treatment with the SCV2-miRNA inhibitors can effectively reduce the virus titer by enhancing IFN signaling in the SARS-CoV-2 virus-infected cell or animal model. Second, it is worth investigating the allelic differential expression of OAS1 in the rs7967461 heterozygote-derived cells after infection with SARS-CoV-2.

In conclusion, our study suggests that SARS-CoV-2 uses its own miRNAs to evade host innate immune response through targeting several genes in the type I interferon signaling pathway. Our study links the functional viral sequence to the host’s susceptible genetic background and opens new directions to investigate the mechanisms underlying the pathogenicity of SARS-CoV-2.

## Data Availability Statement

The datasets presented in this study can be found in online repositories. The names of the repository/repositories and accession number(s) can be found in the article/**Supplementary Material**.

## Author Contributions

YZ performed the computational prediction. ZZ and XG performed the experiments. YZ, ZZ, JS, WQ, and XG analyzed the data. YZ, ZZ, JS, WQ, XG, and YT wrote the manuscript. FX, NS, and YT designed the experiments and revised the manuscript. All authors contributed to the article and approved the submitted version.

## Funding

This study was funded by the National Natural Science Foundation of China (No. 81871287 to YT and No. 31930037 to NS). Open access publication fees were provided by Renji Hospital, Shanghai Jiao Tong University School of Medicine. This work was supported by the National Natural Science Foundation of China (Grant Nos. 81871287 and 31930037) and by the Innovative Research Team of High-Level Local Universities in Shanghai (Grant SSMU-ZDCX20180100).

## Conflict of Interest

The authors declare that the research was conducted in the absence of any commercial or financial relationships that could be construed as a potential conflict of interest.

## Publisher’s Note

All claims expressed in this article are solely those of the authors and do not necessarily represent those of their affiliated organizations, or those of the publisher, the editors and the reviewers. Any product that may be evaluated in this article, or claim that may be made by its manufacturer, is not guaranteed or endorsed by the publisher.
