# Supplementary material for: SARS-CoV-2-Encoded MiRNAs Inhibit Host Type I Interferon Pathway and Mediate Allelic Differential Expression of Susceptible Gene
Source: Front Immunol. 2021 Dec 23;12:767726. doi: 10.3389/fimmu.2021.767726 (PMC8733928; doi:10.3389/fimmu.2021.767726)
Supplement: Supplementary file 1 [file DataSheet_1.zip › 767726_SupMaterial(1)/767726_SupMaterial/Supplementary File6.pdf]

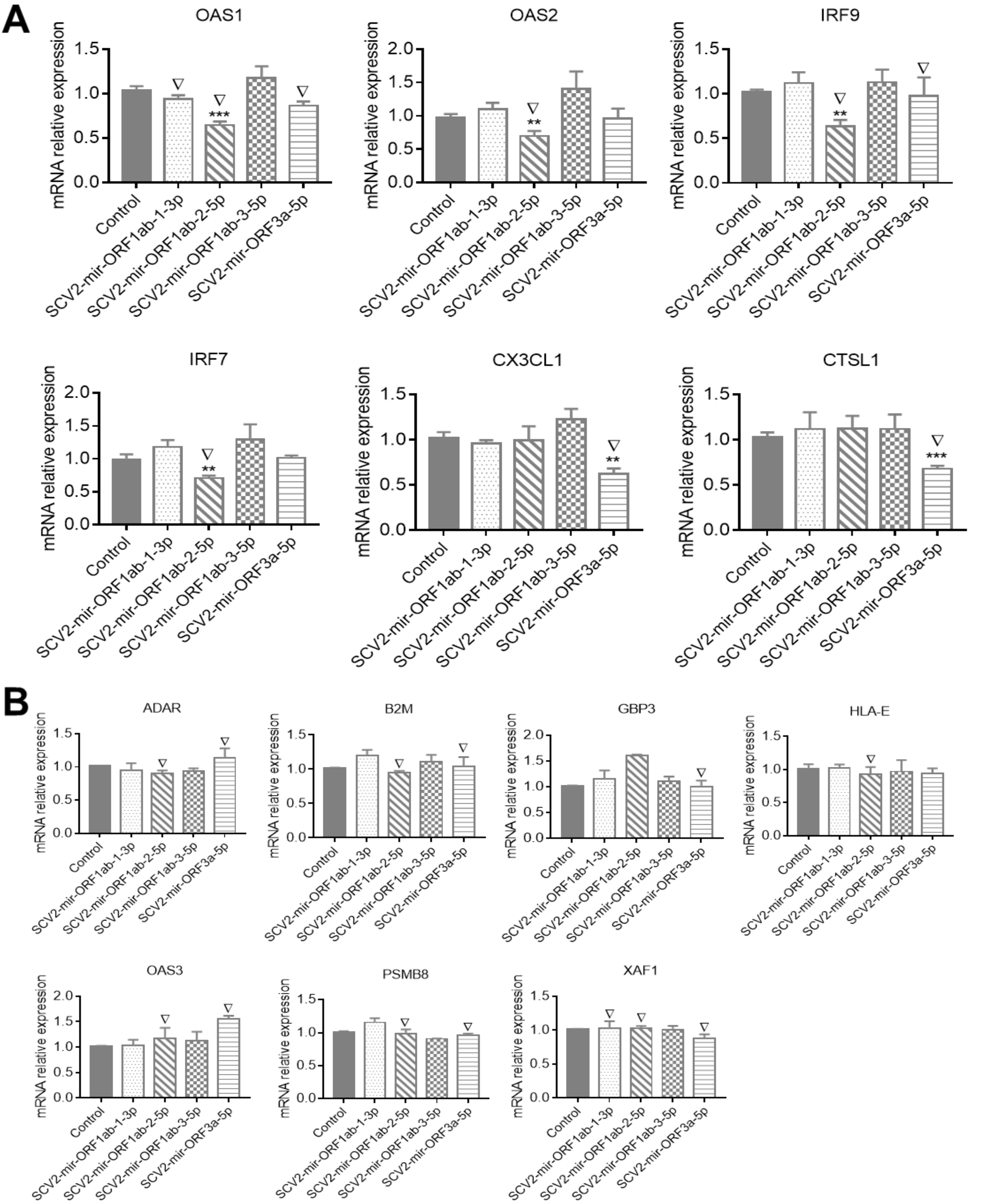

Supplementary File 6. Schematic diagram of RT-PCR quantification of genes targeted by SCV2-miRNAs

A, Quantitative PCR analysis of effectively inhibited genes expression in HEK293T cells 48 hours post-transfection of different SCV2-miRNAs versus control. B, Relative expression level of genes not effectively inhibited was measured in HEK293T cells post-transfection with different SCV2-miRNAs for 48 hours by RT-PCR. The inverted triangle indicates the putative target of SCV2-miRNAs predicted by RNAhybrid. Histograms show fold changes in mRNA expression with respect to the controls after normalization with the housekeeping gene GAPDH. Data are representative of three independent experiments . P-values were analyzed with two-tailed unpaired t-test. $^{**}p < 0.01$, $^{***}p < 0.001$, $^{****}p < 0.0001$.
